# Emerging Function of Prolactin-Inducible Protein—Is This Important Tear Protein Found in Alzheimer’s Disease?

**DOI:** 10.3390/cells15111029

**Published:** 2026-06-03

**Authors:** James Chmiel, Wiktor Gawełczyk, Julia Soczyńska, Jerzy Leszek

**Affiliations:** 1 Faculty of Physical Culture and Health, Institute of Physical Culture Sciences, University of Szczecin, Al. Piastów 40B block 6, 71-065 Szczecin, Poland; 2 Student Scientific Group of Psychiatry, Wroclaw Medical University, Wybrzeże L. Pasteura 10, 50-367 Wroclaw, Poland; wiktor.gawelczyk@student.umw.edu.pl (W.G.);; 3 Department of Psychiatry, Wroclaw Medical University, 50-367 Wroclaw, Poland

**Keywords:** Alzheimer disease, prolactin-inducible protein, biomarker, tear fluid

## Abstract

Alzheimer’s disease is characterized by a chronic, long-term neurodegenerative process and an increasing need for easily accessible biomarkers that enable early diagnosis and disease monitoring. For this reason, tears have attracted growing interest as a potential source of such biomarkers, and prolactin-inducible protein is a candidate tear protein of mechanistic interest whose clinical value remains to be established as a biomarker of Alzheimer’s disease. The literature indicates that prolactin-inducible protein is physiologically present in the lacrimal apparatus. Proteomic studies in patients with Alzheimer’s disease have repeatedly demonstrated decreased levels of prolactin-inducible protein in tears, typically accompanied by reduced concentrations of other proteins associated with normal lacrimal gland function. Although the evidence remains inconclusive, these findings suggest that alterations in prolactin-inducible protein levels may reflect lacrimal gland dysfunction related to neurodegenerative processes, autonomic dysregulation, and inflammation. Nevertheless, the lack of specificity of prolactin-inducible protein for Alzheimer’s disease, as well as the influence of various factors on its concentration, limit its value as a standalone biomarker. The most plausible approach is the incorporation of prolactin-inducible protein into multimarker panels, which could enable improved patient stratification and assessment of lacrimal gland dysfunction in Alzheimer’s disease.

## 1. Introduction

Alzheimer’s disease (AD) is increasingly framed as a long preclinical continuum in which amyloid, tau, synaptic dysfunction, neuroinflammation, and neurodegeneration accumulate for years before dementia is clinically obvious. This biological view has driven intense demand for scalable, low-burden biomarkers to complement (or triage toward) PET imaging and CSF assays—methods that are accurate but relatively invasive, expensive, or logistically constrained. Contemporary biomarker frameworks (e.g., AT(N) logic and related staging concepts) have made it clear that broad screening and longitudinal monitoring will require biofluids that are easier to collect repeatedly in real-world settings [1].

Tears have emerged in that context as a pragmatic “peripheral-but-organ-proximal” biofluid: they are collected quickly, with minimal training, and can be sampled repeatedly with relatively high patient acceptability. Over the last 10–15 years, tear research has matured from cataloging abundant tear proteins to deploying modern mass-spectrometry proteomics, targeted quantification, and increasingly multi-omic workflows (e.g., microRNAs; extracellular vesicles), supported by a parallel methodological literature focused on standardizing collection and processing. Reviews spanning the 2010s to the 2020s describe tear fluid as a rich biomarker source not only for ocular surface disorders but also for systemic diseases, including neurologic disease, because tears integrate inputs from lacrimal gland secretion, conjunctival/corneal epithelium, immune cells, and vascular-adjacent processes [2,3,4,5,6].

The conceptual basis for tears in AD is often presented as an “eye–brain axis”: the eye shares developmental origins and many molecular vulnerabilities with the central nervous system, and ocular tissues can reflect neurodegenerative biology. This idea is widely discussed within the broader umbrella of ocular biomarkers for AD, where retinal imaging (e.g., OCT-derived retinal thickness) is studied as a non-invasive neurodegeneration readout. Although retinal thickness changes are not equivalent to tear biomarkers, this wider ocular-biomarker movement has helped normalize the premise that ocular tissues and fluids may carry measurable signatures of AD pathophysiology and has emphasized careful control of ophthalmic confounders [7,8,9,10,11].

A second, very practical driver is that AD and aging are frequently accompanied by ocular surface dysfunction and dry eye disease (DED)-like changes—conditions known to reshape tear flow, osmolarity, and inflammatory composition. This matters for two reasons. First, tear biomarkers can be biologically informative precisely because they capture barrier/immune remodeling at the ocular surface. Second, it means tear biomarker studies in AD must explicitly confront confounding by DED, meibomian gland dysfunction, medications, and age-related lacrimal changes. Recent syntheses of ocular surface changes in neurological disease explicitly list AD among conditions associated with tear film instability and altered secretion metrics, reinforcing the need for robust clinical phenotyping in biomarker cohorts [12,13,14].

Against this backdrop, several independent clinical studies since the mid-2010s have tested whether AD is associated with a distinctive “tear signature.” The most widely cited early work demonstrated AD-associated alterations in tear flow rate, total protein concentration, and the tear “chemical barrier,” and proposed that combinations of abundant tear proteins could classify AD versus controls with moderate accuracy [15]. Subsequent studies broadened the molecular scope by reporting that tear proteins and microRNAs are differentially expressed across AD/MCI/controls, strengthening the claim that tear fluid can capture disease-linked molecular shifts [16]. More recently, studies have explicitly interrogated neuroinflammation-related tear proteins in mild AD and reported coordinated patterns of up- and down-regulated tear components, positioning tears as a potential window into early inflammatory biology (while still requiring rigorous validation) [17,18].

Finally, a major reason tears remain compelling is that they can, in principle, carry canonical AD analytes in measurable form. In the last several years, multiple groups have investigated tear measurements of amyloid-beta and tau (including phosphorylated tau species and extracellular-vesicle-enriched fractions), motivated by the hope that tears could serve as a minimally invasive screening layer before confirmatory CSF/PET. These directions are still evolving, but their existence underscores why abundant tear proteins—such as lacrimal/ocular surface homeostasis proteins—are being re-examined for relevance to AD staging and inflammatory phenotypes [19,20,21].

## 2. Materials and Methods

Articles relevant for the presented review were identified by conducting searches in PubMed (https://pubmed.ncbi.nlm.nih.gov/), Scopus (https://www.scopus.com/) and Google Scholar (https://scholar.google.com/) databases. The analysis was limited to articles published in English between January 1983 and May 2026. A manual search was also performed using keywords such as “prolactin-inducible protein”, “PIP”, “tears”, “lacrimal gland”, “Alzheimer’s disease”, “tear proteomics”, and “dry eye disease”, as well as their combinations, in order to identify relevant publications. Subsequently, abstracts were screened to select studies meeting the inclusion criteria for this review. The selected studies investigated the tear proteome and PIP expression in the context of lacrimal system physiology, ocular surface diseases, and neurodegenerative processes, including Alzheimer’s disease.

This work is a narrative review rather than a systematic one. A formal systematic synthesis in accordance with the PRISMA guidelines has not been performed, nor has a quantitative meta-analysis been conducted. The synthesis of results presented herein is therefore qualitative in nature. The narrative format was adopted owing to the heterogeneity of the available studies, arising from the use of different methods for the quantification of tear proteins. As a consequence, a rigorous quantitative synthesis is not feasible given the insufficient body of clinical evidence currently available.

The review included original research articles (mainly tear proteomic studies and studies of tissue PIP expression), supplemented by selected clinical studies providing pathophysiological context for the proteomic observations, as well as mechanistic and systematic reviews, with particular attention to studies providing primary quantitative or functional data on PIP. Conference abstracts without full-text access, non-English-language publications, and descriptive publications lacking empirical data were excluded. The broad time window (January 1983–May 2026) reflects two distinct search objectives: early publications from 1983 to 2010 were included to document the fundamental biology of PIP (cloning, structure, transcriptional regulation), whereas studies addressing the role of PIP in Alzheimer’s disease and in ocular conditions originate from a narrower, more recent window encompassing primarily the years 2015–2026.

Priority was given to studies in which proteomic data were publicly available in repositories, as this enables independent reanalysis of the results. Such an approach contributes to enhancing the methodological rigor of research investigating PIP in AD.

## 3. PIP Fundamentals: Gene, Protein, Expression Pattern

Prolactin-inducible protein (PIP) is a human secreted glycoprotein that has accumulated a notably complex identity across decades of research because it was independently discovered in different secretions and clinical settings. In breast pathology it became widely known as GCDFP-15 (gross cystic disease fluid protein-15), historically associated with apocrine differentiation and used as an immunohistochemical marker in subsets of breast carcinomas; in reproductive biology it was purified from seminal plasma as gp17 and described as a secretory actin-binding protein (SABP); and in salivary research it appears as EP-GP (extraparotid glycoprotein), later shown to be identical to GCDFP-15/PIP. This “many names for one protein” history is not just nomenclature trivia: it captures a consistent biological theme—PIP is enriched in secretory/apocrine-like epithelia and is released into multiple mucosal fluids where it can participate in surface defense and protein–protein interactions. Modern curated resources unify these aliases under the approved gene symbol PIP (HGNC:8993) and cross-reference historical synonyms (including BRST-2, GCDFP-15, GCDFP15, GPIP4, SABP) [22,23].

At the gene level, PIP is a protein-coding locus on chromosome 7. Contemporary genome annotations place the gene on the forward strand in the 7q region with stable coordinates in current assemblies, and Ensembl summarizes the locus (ENSG00000159763) with curated synonyms and orthology across vertebrates. A classic molecular genetics paper from the early 1990s established core structural features that still anchor how the field discusses PIP regulation: the entire gene was reported to be ~7 kb long, organized into four exons, and hormonally responsive at the transcriptional level. In that work, both androgen and prolactin increased transcription of the PIP gene (as assessed by nuclear run-on experiments), whereas effects on RNA stability were not supported—an early indication that tissue- and hormone-specific transcriptional control is central to the PIP story [23,24]. This hormonal logic has been reinforced and extended by subsequent literature emphasizing that PIP/GCDFP-15 is part of endocrine-responsive secretory programs in breast epithelium and other exocrine organs, while also noting that regulation can be tissue-specific (e.g., not all sites mirror breast cancer cell line behavior) [25,26]. Standard gene resources (NCBI Gene: 5304; KEGG hsa:5304) provide consolidated entry points that link the locus to RefSeq proteins and function-oriented literature notes (GeneRIFs), reflecting how PIP has become embedded in diverse research areas ranging from secretion biology to immunity and cancer [27,28].

At the protein level, UniProt (UniProtKB release 2026_01) curates PIP as a secreted protein bearing an N-terminal signal peptide, consistent with synthesis in the secretory pathway and release into extracellular fluids. The canonical human sequence is small (UniProt annotates the reviewed human protein as 146 amino acids including signal peptide), which historically led to “15–17 kDa” descriptions that vary depending on maturation state and glycosylation. A recurring point in older biochemical literature is that PIP’s apparent molecular mass can differ substantially across fluids and tissues because it is a glycoprotein with disulfide bonds and post-translational modification heterogeneity. UniProt explicitly catalogs modification features (including glycosylation/disulfide annotations), and glycoprotein-focused knowledgebases cross-link these features to glycosylation/structure records. This is important for both function and measurement: tissue-specific glycoforms may alter binding interactions (e.g., with proteins or microbes) and can influence immunoassay recognition—practical considerations when comparing PIP levels across cohorts or biofluids such as tears [29].

A striking advance in “PIP fundamentals” came from structural biology: PIP was crystallized in complex with zinc-alpha-2-glycoprotein (AZGP1/ZAG) purified from human seminal plasma, and the complex structure is archived as PDB 3ES6 (superseding an earlier deposited entry). The RCSB record frames this as a novel stable complex and provides a concrete structural foundation for a theme that runs through decades of experimental work—PIP often behaves as an extracellular interaction partner in secretions rather than a lone effector molecule. In other words, PIP’s “function” has frequently been inferred from what it binds, and having solved complexes supports the plausibility that at least some interactions are specific and structurally constrained rather than nonspecific adsorption [30].

Mechanistically, several major functional hypotheses have emerged from biochemical and immunological studies. First, in the seminal plasma literature, PIP (as gp17/SABP) was associated with actin binding, aligning with the “secretory actin-binding protein” label that persists in some databases and reviews. Second, in the salivary field, EP-GP (identical to GCDFP-15/PIP) was shown to bind bacteria in vivo, with a well-cited study reporting selective binding to multiple oral and non-oral bacterial species—an observation that has been repeatedly discussed as consistent with a role in mucosal host–microbe interactions [31,32]. Third, an immunology-linked line of work reported that gp17/PIP can bind CD4, and a *Biochemistry* paper mapped CD4-binding determinants within the molecule, fueling hypotheses that PIP may modulate T-cell signaling or apoptosis under certain conditions [33,34]. Fourth, a particularly influential cancer-associated claim proposed that GCDFP-15/PIP exhibits aspartyl protease activity (“a novel aspartyl proteinase”), which—if operative in vivo—could connect PIP to extracellular matrix remodeling, barrier effects, or processing of secreted substrates [35]. Later literature has continued to cite and explore these themes in disease contexts (especially breast cancer and epithelial biology), while also reflecting that functional effects can be context-dependent and may vary with tissue source, molecular complexing, and glycoform composition [25,33,36].

PIP’s expression pattern is unusually consistent across “old” and “new” data streams: it is preferentially produced by secretory epithelia and enriched in exocrine organs. The early gene-structure/regulation paper already emphasized expression in normal exocrine tissues such as sweat, salivary, and lacrimal glands (in addition to benign and malignant breast tissue) [23]. Modern curated protein records retain the same theme, explicitly noting expression in exocrine tissues including lacrimal, salivary, and sweat glands. Tissue atlases provide a more systematic view: the Human Protein Atlas (HPA) highlights selective cytoplasmic expression in salivary glandular cells and breast glands, and its tissue-specific pages integrate RNA-seq evidence from HPA/GTEx and related datasets to summarize baseline expression patterns. Cross-database integrators similarly converge on a “secretory tissue” signature for PIP, listing broad expression across many tissues but with strong enrichment in sites like seminal vesicle/salivary-type glands and other secretory epithelia (as captured in expression compendia such as Bgee and in target profile aggregators like Open Targets). Importantly for tear biology and for the question of whether PIP is a plausible tear biomarker in neurodegeneration, multiple sources link PIP/GCDFP-15 expression to lacrimal-associated tissues: UniProt explicitly lists lacrimal glands among exocrine expression sites, and pathology-oriented discussions note expression in accessory lacrimal glands alongside salivary serous cells, reflecting a broader ocular adnexal secretory program [37,38,39].

## 4. PIP as a Tear Protein: Ocular Surface and Lacrimal Apparatus Evidence

A central prerequisite for interpreting any disease-associated change in tear PIP is to establish, as rigorously as possible, (i) that PIP is a bona fide constituent of the normal human tear film, (ii) which ocular adnexal tissues plausibly supply it, and (iii) whether its abundance varies with common ocular surface states (dry eye, inflammation, epithelial remodeling) that could confound neurodegeneration studies. Over several decades, the “tear PIP” question has been addressed by convergent lines of evidence: classic immunohistochemistry of apocrine-like tissues (including eyelid glands), modern ocular surface and lacrimal gland expression mapping, repeated identification of PIP in unbiased tear proteomes collected by multiple methods, and targeted quantification (ELISA/Western-type approaches) in defined clinical cohorts. What emerges is a consistent picture: PIP is not an occasional contaminant but a recurrent tear constituent whose levels appear sensitive to lacrimal–ocular surface secretory programming and to inflammatory/epithelial remodeling processes that are themselves common in aging and systemic disease.

The earliest tissue-level hints that the “GCDFP-15/PIP family” might be relevant to ocular adnexa came from pathology-era immunolocalization studies of GCDFP-15, which emphasized its expression in apocrine glands in multiple sites, including the eyelid. In a widely cited 1983 paper that helped establish GCDFP-15 as an apocrine marker, immunoperoxidase staining localized GCDFP-15 in apocrine glands of the axilla, vulva, ear canal, and importantly eyelid structures, creating an anatomical bridge between this protein family and ocular adnexal secretory tissues [40]. While that work predated modern tear proteomics, it made biologic sense that a small secreted apocrine-associated glycoprotein could appear in ocular surface secretions, especially given the mixture of lacrimal and eyelid gland contributions that form the tear film.

Direct lacrimal/salivary gland biology strengthened that inference. In the late 1990s, Mirels and colleagues examined GCDFP-15/PIP expression patterns in salivary and related exocrine glands and highlighted that rodent PIP transcripts had been demonstrated in adult submandibular and lacrimal glands; their work further characterized rat PIP expression in neonatal and adult glandular tissues, reinforcing that PIP is embedded in exocrine secretory programs that include the lacrimal gland lineage [41]. Developmental biology studies in the early 2000s extended this by mapping the endogenous expression pattern of the mouse homolog in embryonic/postnatal development, supporting the idea that PIP-family expression is developmentally regulated in secretory epithelial lineages rather than being an artifact of adult pathology [42]. These older glandular-expression datasets are relevant to tears for a simple reason: proteins that are both (a) secreted and (b) encoded by lacrimal/acinar epithelial programs are precisely the proteins that reliably recur in tear proteomes.

The modern anchor evidence for PIP as a tear protein, however, comes from explicit studies of the human lacrimal apparatus and ocular surface. A key 2022 paper used an integrated approach spanning gene/protein expression in ocular tissues and direct testing of human tear fluid. The authors reported that PRL, PRLR, and PIP are detectable in tissues of the lacrimal apparatus and ocular surface, but that PIP—not prolactin itself—is present in tears, and they linked this observation to tear film quality and evaporative DED mechanisms [43]. In the same research program, an ARVO/IOLVS abstract (2019) focused on the “prolactin → PIP → AQP5” axis in the human lacrimal gland and corneal epithelium, analyzing how prolactin influences PIP and aquaporin-5 (AQP5) expression—an especially important point because AQP5 membrane localization and lacrimal gland secretory physiology are core determinants of tear volume and composition [44]. Together, these studies establish a mechanistic plausibility chain: PIP is not merely “present somewhere near the eye,” but is positioned within endocrine/secretory signaling that plausibly affects tear film properties, and it is empirically detectable as a soluble tear component.

Tissue localization within the ocular surface/lacrimal unit further supports plausible sources of tear PIP beyond the main lacrimal gland alone. The 2022 study included immunohistochemical localization across multiple ocular tissues; while many figures are widely re-shared in derivative contexts, the core claim is that PIP is expressed in the lacrimal gland, corneal epithelium, and eyelid regions associated with tear film maintenance [43]. This fits the broader “lacrimal functional unit” concept: the tear film is a composite of aqueous secretion (lacrimal), mucins and immune factors (conjunctiva/ocular surface), and lipid layer contributions (meibomian glands), with significant cross-talk via neural and inflammatory pathways. Therefore, even if PIP is primarily secreted by lacrimal acinar/ductal epithelia, its measured tear abundance could be shaped by ocular surface inflammation, evaporative stress, and eyelid gland dysfunction that alter secretion, dilution, and proteolysis.

Independent of tissue mapping, the strongest practical evidence that PIP is a real tear constituent is its repeated identification in unbiased tear proteomic catalogs across methods, instruments, and clinical contexts. Tear proteomics has evolved from early pooled-sample profiling to deep, quantitative workflows capable of identifying well over a thousand proteins from Schirmer strips or microliter volumes. Studies explicitly comparing Schirmer strip sampling (high yield, but potential ocular surface cell contribution) with capillary tube collection (cleaner aqueous tears but lower volume) have shown that collection method impacts the proteome, yet a core set of “high-confidence tear proteins” persists across workflows [45,46,47,48]. Tear proteomics method papers emphasize that robust biomarker candidates should be detectable under multiple collection conditions and across repeated sampling, because strip-derived protein patterns can be influenced by conjunctival/corneal epithelial contact, reflex tearing, and sample handling [45,46,47]. Within this methodological landscape, PIP is frequently identified and is sometimes highlighted among abundant tear proteins in tear-proteome reviews or overviews [49,50].

Clinical tear proteomics further demonstrates that PIP behaves like a regulated tear component rather than a random background protein. In thyroid eye disease, for example, a 2018 *Scientific Reports* paper proposed that tear proteins including PIP (together with S100A4) could serve as potential biomarkers, implying not only detectability but also disease-linked modulation of tear PIP in an inflammatory orbital/ocular surface condition [51]. In keratoconus, a 2014 PLOS ONE study identified GCDFP-15/PIP as an independent discriminator between keratoconus and controls, reporting lower intensity in keratoconus tears and downregulation in vitro under TGF-β stimulation; later clinical work summarized and extended these observations across tears, plasma, and saliva, reinforcing that tear PIP can be systematically altered in a corneal remodeling disorder [52,53]. While keratoconus is not directly related to neurodegeneration, it is highly informative for AD tear-biomarker interpretation because it demonstrates that PIP is sensitive to epithelial remodeling and cytokine pathways—exactly the kinds of confounders that can accompany aging and systemic inflammation.

DED is an especially important confounder and mechanistic context because it is common in older adults and because DED fundamentally reshapes tear protein composition through altered secretion, evaporation, inflammation, and epithelial stress. Multiple tear proteomics studies (including early iTRAQ-based quantitative analyses and later deep MS surveys) show that DED subtypes are associated with consistent shifts in tear proteins, and at least some datasets and secondary analyses highlight PIP as among the proteins that can change with dry eye severity or subtype [13,50,54]. The 2022 ocular-surface/lacrimal unit paper explicitly connected PIP to evaporative dry eye disease, placing tear PIP within a physiologic narrative of tear film quality rather than treating it as a purely diagnostic marker [43]. From a biomarker-development standpoint, these observations imply that any AD-associated signal in tear PIP must be interpreted against a background where tear PIP is already known to respond to ocular surface stress and inflammation—meaning that careful phenotyping (tear breakup time, meibomian function, osmolarity, ocular surface staining, Schirmer test, medication use) is not optional if one wants to claim AD specificity.

Mechanistic plausibility for why tear PIP would be sensitive to systemic autoimmune/exocrine disease also comes from the large body of work on Sjögren’s syndrome (SS) and exocrine gland dysfunction, which frequently centers on AQP5 mislocalization and secretory hypofunction in lacrimal and salivary glands. Reviews on AQP5 dysregulation in SS and SS-related dry eye explicitly discuss PIP as an AQP5 partner and potential regulator of AQP5 trafficking, positioning PIP within a protein–protein interaction network that directly influences exocrine secretion physiology [55,56,57]. Molecular-interaction work has even attempted to model AQP5–PIP interactions directly, highlighting that PIP is not merely co-expressed but is hypothesized to interact with or influence the machinery that controls apical water channel localization [56,57]. Although much of this work is salivary-focused, it is repeatedly motivated by the parallel between salivary and lacrimal gland secretory biology and the shared clinical phenotype of sicca [57,58]. The implication for tears is straightforward: if PIP participates in the regulatory logic of AQP5 placement and exocrine secretion, then tear PIP could shift not only in ocular surface diseases but also in systemic conditions with exocrine dysfunction—and potentially in neurodegenerative states where autonomic and inflammatory changes influence gland function.

The question of whether PIP is part of a stable “core tear proteome” has also become easier to address as tear proteomics has matured and as the community has begun to curate tear protein resources. Mass-spectrometry-centric reviews emphasize that, despite variability from sampling and processing, there is a reproducible subset of abundant tear proteins and a long tail of lower-abundance proteins that appear depending on depth and method [46,47,49]. A notable recent development is the emergence of dedicated tear proteomics resources, including a 2026 report describing a tear fluid database intended as a reference website integrating protein and clinical data across studies [59]. Although such databases are still new and will evolve, their very creation reflects the field’s recognition that tear proteins—including candidates like PIP—should be evaluated in the context of cross-study reproducibility and methodological provenance rather than isolated single-cohort findings.

Taken as a whole, the ocular surface and lacrimal apparatus evidence supports three highly relevant conclusions for an AD-focused tear biomarker narrative. First, PIP is demonstrably produced within the lacrimal/ocular adnexal secretory ecosystem and is directly detectable in tears, with explicit modern studies arguing that it contributes to tear film quality and is present independently of prolactin itself. Second, PIP behaves like a regulated tear component: it is repeatedly identified in tear proteomes and shows disease-linked modulation in multiple ocular surface disorders (thyroid eye disease; keratoconus; dry eye), indicating sensitivity to inflammation, epithelial remodeling, and secretory state. Third, because PIP is tied to exocrine gland physiology (including AQP5-related secretory mechanisms discussed in SS and exocrine dysfunction literature), changes in tear PIP could plausibly reflect a broader systemic or neuroimmune influence on gland function—precisely the kind of “bridge biology” that makes tears attractive in AD research but also demands rigorous control of ocular surface confounding.

## 5. Evidence That PIP Changes in AD Tears

The evidence base for PIP changing in AD tears is still relatively compact compared with the CSF/plasma biomarker literature, but it is unusually coherent in direction across independent approaches: when PIP is explicitly measured or emerges among “lacrimal-gland/tear-specific” proteins, it is repeatedly reported as lower in AD tears than in cognitively healthy controls. The most frequently cited primary source for a quantitative PIP effect in AD tears is the 2016 targeted-proteomics study by Kalló et al. (PLOS ONE), which was designed not as a deep “discovery tear proteome” but as a tear chemical barrier study with subsequent candidate biomarker modeling. In that work, the authors first observed altered tear flow/protein patterns and gel band differences between AD and controls, then used LC–MS/MS on excised gel bands to identify proteins showing visible differences, and finally applied selected reaction monitoring (SRM) to quantify ten preselected abundant tear proteins across 37 tear samples (14 AD patients, 9 controls, both eyes sampled in many participants). Within this SRM panel, PIP was significantly decreased (log2 fold change—0.66) in AD tears alongside other lacrimal-gland-linked proteins (lipocalin-1, lactotransferrin, lacritin, lysozyme-C), while dermcidin was increased. Crucially, their mixed-effects variance analysis reported PIP log2 fold change ≈ −0.66 with adjusted *p* < 0.0001, making PIP one of the strongest statistical signals in their targeted set. It is noteworthy that an additive effect of proteins was observed in terms of test performance. The AUC was 0.6; however, when two or more proteins were combined, it exceeded 0.7 [15].

Because tear film composition is sensitive to reflex tearing, sampling method, ocular surface inflammation, and medication use, it matters that Kalló et al. framed the PIP decrease not as an isolated marker but as part of a cluster of downregulated lacrimal-secreted defense proteins, interpreting the pattern as consistent with lacrimal gland dysfunction in AD. They explicitly note that the downregulated proteins are expressed by lacrimal glands and discuss reduced corneal sensitivity/abnormal tear function in neurodegenerative disease as a plausible physiologic context for tear composition changes. From a biomarker-science perspective, this “pattern logic” is of considerable importance. Although the levels of an individual tear protein may fluctuate for numerous reasons, coordinated alterations across multiple tear- and lacrimal gland-associated proteins suggest that Alzheimer’s disease involves a genuine shift in the overarching secretory program (autonomic, inflammatory, endocrine, or glandular). In this framework, PIP may potentially serve as one of the biomarkers reflecting this process.

A second, more recent and methodologically distinct line of evidence comes from a 2025 *Journal of Molecular Neuroscience* paper by Kärkkäinen et al., explicitly motivated by the hypothesis that tear proteins related to neuroinflammation could provide early, non-invasive biomarker candidates in mild AD. In this study, tear fluid proteomics was performed with label-free quantification (PEAKS, with stated peptide/protein filters and FDR controls), and the authors report 14 proteins with altered expression linked to inflammation/AD pathogenesis in a cohort of 53 participants (34 cognitively healthy controls vs. 19 with clinically mild AD dementia; CDR 0.5–1). In their results, PIP and SCGB2A1 were the only significantly downregulated proteins in that 14-protein inflammatory set (with several acute-phase/immune proteins increased) (*p* ≤ 0.05). They also emphasize that PIP is among “common tear-specific proteins” and interpret the downregulated tear-specific proteins (including PIP) as again consistent with lacrimal gland dysfunction and consistent in direction with earlier tear studies [17]. This is valuable as replication-in-spirit even though the overall study question (neuroinflammation-linked proteins) and analytic workflow differ from Kalló’s targeted SRM panel: both converge on lower tear PIP in AD as part of a broader lacrimal/tear homeostasis signature.

It is also informative that the 2025 neuroinflammation paper tries to explicitly reduce ocular confounding by recruitment exclusions (e.g., excluding diabetes and overt eye disease) and by reporting ocular surface exam characteristics, because a major critique of tear biomarkers in systemic disease is that “common” ocular surface problems in older adults could drive the signal. While no observational design can eliminate all confounding, this explicit attention to ocular factors supports the plausibility that the observed PIP decrease is not simply dry eye noise—especially since the direction matches Kalló et al.’s earlier results and the same study highlights that multiple lacrimal-produced proteins trend down together.

Beyond these two core “PIP is lower” studies, the broader tear-in-AD literature adds supportive context in two ways. First, AD tear biomarker work has expanded into canonical AD molecules (amyloid and tau), showing that tears can carry CNS-relevant biomarker signals and that tear changes can correlate with neurodegeneration severity, strengthening the general rationale that tear composition can reflect neurodegenerative states rather than being purely ocular. For example, Gijs et al. (2021, *Scientific Reports*) report associations between tear tau (and amyloid/tau status) and neurodegeneration, and—importantly for PIP—this paper summarizes earlier tear protein work as having observed differences in PIP among other tear proteins, embedding PIP into the recognized “tear protein altered in AD” set even when PIP is not the primary analyte [60]. Second, multiple reviews focusing on tears as biomarkers in neurodegeneration repeatedly cite PIP among tear proteins reported as altered in AD, generally aligning with the “downregulated lacrimal proteins” narrative and emphasizing the need for validation and clinical standardization [12,61,62].

A different form of “evidence” that is increasingly important in modern biomarker science is the existence of public proteomics datasets underlying published tear-in-AD studies. Tear proteomics from AD/MCI/control cohorts has been deposited into community repositories, enabling independent reanalysis and cross-study comparisons (even if formal meta-analyses remain rare). For example, ProteomeXchange/PRIDE lists a dataset titled “Proteins present in tears from control, MCI and Alzheimer disease patients”, explicitly describing LC–MS/MS tear protein evaluation in MCI and AD, and linking to the PRIDE project record. Even when individual proteins are not foregrounded in an abstract, the availability of raw/processed proteomic data means PIP-direction claims can, in principle, be checked and re-modeled across cohorts and pipelines—an important step toward determining whether PIP is a stable AD-associated tear signal or a cohort-specific finding. In parallel, the emergence of tear proteomics reference resources (e.g., newly launched tear proteome databases) is beginning to make “is this protein a reproducible tear constituent, and how variable is it across studies?” a practical question rather than an anecdotal one—relevant because PIP is a robust tear protein, but its baseline variance and sensitivity to ocular surface states directly affect biomarker reliability [17,59,63].

However, a careful reading of the AD tear PIP evidence also highlights the current limitations and what “changes in PIP” can realistically mean today. First, replication is directionally consistent but still sparse: the strongest quantitative evidence is Kalló et al.’s SRM (targeted, high-precision for the chosen proteins) and Kärkkäinen et al.’s label-free discovery/functional subset analysis (broader but potentially more sensitive to missingness and normalization choices). Second, tear PIP is biologically tied to lacrimal/ocular surface secretory physiology, and PIP is known to vary in ocular surface disorders and inflammatory remodeling contexts.

We emphasize that any association between PIP and AD must be interpreted as either (a) a relatively direct consequence of AD-linked autonomic/neuroimmune changes on the lacrimal functional unit, or (b) an indirect association mediated by higher rates of subclinical ocular surface dysfunction in AD cohorts (reduced blink, altered corneal sensitivity, medication burden, hydration/nutrition differences, etc.). The best AD tear studies increasingly acknowledge these issues, and some designs incorporate ophthalmic characterization to mitigate them, but this remains a central challenge for translating a PIP shift into an AD-specific diagnostic. Third, because PIP changes appear as part of a multi-protein lacrimal signature, it may perform better as one feature in a panel (where multiple lacrimal defense proteins move together) rather than as a stand-alone test. Kalló et al. explicitly explored multivariate ROC combinations and found that combining proteins improved discrimination compared with single markers; while their “best-performing” combinations varied, PIP appeared in multiple combinations, supporting the idea that PIP may add information in combination even if it is not the single strongest discriminator across cohorts.

In summary, in relation to the question “does PIP change in the tears of patients with AD?”, when PIP is directly quantified or identified among lacrimal-gland-specific proteins, its levels are repeatedly reported as decreased in the tears of patients with AD. This reduction is typically accompanied by decreased levels of other lacrimal defense proteins (lipocalin-1, lysozyme, lactoferrin, lacritin), suggesting a phenotype consistent with lacrimal functional unit (LFU) dysfunction and impaired secretory activity, rather than a change uniquely specific to PIP itself. Notably, given the potential for confounding factors, these findings have also been reproduced in studies that implemented recruitment exclusion criteria to mitigate the influence of ophthalmic comorbidities. The key outstanding question in the emerging framework of “PIP function in AD” therefore concerns not only whether PIP is altered, but whether such alterations are specific to AD (as opposed to changes associated with aging, dry eye disease, systemic inflammation, or other forms of dementia), and whether they correlate with disease stage, progression, and/or positivity of established AD biomarkers in longitudinal cohorts. This is precisely the rationale underlying ongoing prospective initiatives (e.g., TearAD and related tear biomarker validation studies), which aim to integrate tear-derived signals with established neurodegeneration markers and clinical disease trajectories. When the current quantitative evidence is considered collectively, interpretive caution is warranted. First, the magnitude of the PIP effect is modest and is reported on non-comparable scales: a targeted-SRM log2 fold-change of approximately −0.66 [15] cannot be directly equated with the label-free downregulation reported by Kärkkäinen et al. [17], because the quantification platforms, normalization strategies and cohorts differ. Second, the lower PIP level in patients with AD is directional rather than quantitative in nature. The results of both studies do not yield consistent estimates of effect size, and the small, heterogeneous cohorts preclude a formal meta-analytic synthesis. A third argument concerns the limited clinical relevance of the signal. The best-performing diagnostic strategy based on the results of Kalló et al. [15] (lipocalin-1 + dermcidin + lysozyme-C + lacritin; 81% sensitivity, 77% specificity) does not include PIP; rather, the authors of that study describe PIP as one of several components contributing to multi-protein biomarker panels, and not as a single biomarker with stand-alone diagnostic value. The key tear-fluid studies relevant to PIP alterations in Alzheimer’s disease are summarized in Table 1. Tear biomarker candidates in AD are presented in Table 2.

## 6. Why Might PIP Be Altered in AD? Mechanistic Hypotheses (With Testable Predictions)

The repeated observation that tear PIP decreases in AD could be interpreted through the physiology of the LFU—the integrated system in which the ocular surface (cornea/conjunctiva), lacrimal glands (main and accessory), eyelids/meibomian glands, and their sensory–autonomic innervation act as a tightly coupled homeostatic circuit to regulate tear volume and composition. LFU physiology is not an abstract concept: it is the reason tear protein concentrations can shift when corneal nerves degenerate, when parasympathetic tone changes, when lacrimal gland acini inflame, or when epithelial barrier stress increases. Reviews of tear film complexity and neural regulation detail how parasympathetic, sympathetic, and sensory pathways regulate secretion of water/electrolytes and proteins from the lacrimal gland and ocular surface, and TFOS DEWS II explicitly frames dry eye and tear homeostasis around LFU feedback loops and their disruption by inflammation, neuropathy, and autonomic blockade [64,65,66]. Within this framework, PIP may potentially be altered in Alzheimer’s disease through several non-mutually exclusive pathways, each of which suggests testable predictions. It should be emphasized that the mechanisms outlined below serve only as hypothetical interpretive frameworks, derived from indirect evidence, cross-sectional studies, and data not directly related to the visual system. To date, none of these mechanisms have been causally demonstrated to account for the variability of tear PIP levels in patients with AD. The “testable predictions” presented alongside each mechanism have been included by us precisely because of the absence of proven causal relationships.

A first plausible mechanism is autonomic and cholinergic dysregulation leading to lacrimal hyposecretion and “tear-specific protein” depletion. Tear secretion is strongly driven by parasympathetic inputs (acetylcholine and VIP), with sympathetic modulation and sensory feedback from trigeminal afferents; disruption of any limb can reduce basal tear production and alter the protein mixture delivered to the tear film [64,65,66]. AD is increasingly recognized to involve dysautonomia, including reduced parasympathetic activity measurable by heart-rate variability (HRV) and other autonomic tests; reviews and newer clinical studies support parasympathetic impairment and sympathetic predominance in AD cohorts [67,68,69]. If parasympathetic drive to the lacrimal gland is reduced (whether due to central autonomic network pathology, cholinergic deficits, medication effects, or brainstem involvement), one might expect reduced aqueous secretion and reduced delivery of lacrimal-gland-enriched proteins, producing the exact pattern reported in tear AD studies where several lacrimal defense proteins trend down together [15,17,70]. This hypothesis also aligns with clinical ocular findings in AD and neurodegenerative disease more broadly: reduced corneal sensitivity, corneal nerve abnormalities, and abnormal tear function have been reported and reviewed, all of which can weaken afferent LFU feedback and blunt reflex tearing and secretion [11,62,71,72]. Testable predictions: (1) tear PIP should correlate with objective tear production (Schirmer/phenol red thread), tear osmolarity, and other lacrimal-derived proteins (e.g., LCN1, lactoferrin, lysozyme, lacritin) within the same individuals; and (2) measures of autonomic function (HRV indices, orthostatic measures) should predict lower PIP independent of age and ocular surface disease severity.

A second mechanism is more molecularly specific: PIP–AQP5 coupling and altered water-channel trafficking in exocrine glands. A long-standing line of work in SS and exocrine gland biology links impaired tear/saliva secretion to abnormal localization/trafficking of AQP5 in acinar cells. Multiple sources—spanning classic lacrimal gland studies through modern reviews—describe that PIP can bind the AQP5 C-terminus and promote AQP5 trafficking to the apical membrane; in a mouse model, disruption of this interaction is associated with aberrant AQP5 distribution, and Ohashi et al.’s lacrimal gland work explicitly proposed that correcting aberrant PIP–AQP5 binding could normalize AQP5 trafficking [56,58,73]. This may be relevant in AD because even modest chronic reductions in lacrimal secretory competence—whether driven by autonomic dysregulation, inflammaging, or epithelial stress—might plausibly reduce PIP secretion and could be associated with disturbed AQP5 localization, potentially contributing to aqueous-deficient and evaporative components of tear dysfunction. Modern ocular-surface work has placed PIP in the lacrimal apparatus and tear film and explicitly connected it to tear quality, while independent AQP5 trafficking reviews and mechanistic papers continue to highlight PIP as a candidate AQP5 trafficking regulator in exocrine glands [43,55,57,73]. Testable predictions will be that (1) in AD lacrimal tissue (or relevant models), PIP levels should covary with AQP5 apical membrane localization and with secretagogue-induced tear flow; (2) tear PIP should track with tear volume measures more strongly than with purely ocular-surface-derived inflammatory markers; and (3) interventions that increase lacrimal secretion (neural stimulation, muscarinic agonist responsiveness) should increase PIP and normalize a “tear-specific protein” signature if the pathway is causal.

A third proposed mechanism is inflammaging/oxidative-stress-driven lacrimal gland remodeling with secondary depletion of protective secreted proteins. Aging itself produces structural and functional lacrimal gland decline characterized by acinar atrophy, fibrosis, immune infiltration (including CD4+ T cells and myeloid cells), oxidative damage markers, and reduced stimulated and basal tear secretion, as shown in influential mouse work and expanded in later mechanistic aging studies [14,74,75]. AD adds a systemic inflammatory and neuroinflammatory milieu on top of aging biology, and recent tear proteomics work in mild AD highlights a pattern compatible with “tear-specific proteins down, inflammatory/acute-phase proteins up,” which is consistent with a glandular secretory program being suppressed while inflammatory proteins become relatively enriched in the tear film [12,15,17]. In such a scenario, PIP could be downregulated because secretory epithelial differentiation and export are impaired (or because acinar cell populations are reduced), rather than because PIP is directly tied to amyloid/tau. Testable predictions will be that (1) tear PIP should inversely correlate with local ocular surface inflammatory signals (e.g., tear cytokines, S100 proteins, acute-phase proteins) and with markers of oxidative stress where measured; (2) lacrimal imaging or biopsy (when available) should show stronger inflammatory/degenerative change in those with lowest PIP; and (3) PIP should show a graded decline with age in controls, with a left-shifted (worse) distribution in AD.

A fourth mechanism focuses on ocular surface neurotrophic changes and altered LFU sensory feedback. The LFU depends on corneal and conjunctival sensory inputs; damage to corneal nerves reduces reflex tearing and can also change epithelial immune tone and dendritic cell density. Multiple ocular–AD reviews and clinical studies describe reduced corneal sensitivity and corneal nerve alterations in AD and other neurodegenerative diseases, and experimental work indicates that tauopathy can impair corneal nerve maintenance and regeneration, providing a plausible mechanistic bridge from neurodegeneration to ocular surface neurotrophic dysfunction [11,71,72]. When sensory drive is reduced, basal tear secretion may decline and protein delivery patterns may shift, which may lead to lower concentrations of lacrimal gland products such as PIP. Testable predictions will be that (1) PIP should correlate with corneal esthesiometry measures and corneal nerve fiber density (confocal microscopy); (2) PIP reduction should be strongest in AD subgroups with the greatest corneal nerve loss; and (3) if corneal sensory input is experimentally enhanced (e.g., trigeminal neurostimulation used clinically for dry eye), PIP should rise alongside improved tear metrics.

A fifth mechanism is endocrine regulation and sex-linked effects on exocrine PIP expression, which may interact with AD risk and aging. PIP is classically hormone-responsive (prolactin and androgens in various tissues), and lacrimal gland work has described tissue-specific hormonal effects on PIP expression (including differences between glands and species) [41,43,44]. Because endocrine axes and sex hormones change with aging—and endocrine dysregulation is common in older adults—PIP could be reduced in AD if endocrine changes (or endocrine medication patterns) differ systematically between cohorts. Testable predictions will be that (1) PIP differences between AD and controls should remain after adjusting for sex, age, endocrine status/therapy, and dry eye severity; (2) stratified analyses should reveal whether the PIP signal is stronger in one sex or in specific hormonal states; and (3) in longitudinal cohorts, within-person PIP change should correlate with endocrine biomarkers if this pathway is significant.

A sixth mechanism is innate defense and microbiome-related remodeling, where PIP changes reflect shifts in ocular surface host defense rather than secretion volume per se. PIP has been repeatedly characterized as a multifunctional secreted protein with bacterial binding and immunomodulatory activity, and modern work continues to investigate its antibacterial effects [33,76,77]. If AD is associated with altered ocular surface immunity (through systemic inflammation, reduced blink, altered tear dynamics, or medication effects), the ocular surface microbial community and immune set point could shift, and tear PIP could be downregulated as part of a broader remodeling of antimicrobial protein networks. Testable predictions will be that (1) PIP should covary with other antimicrobial tear proteins and with ocular surface inflammatory markers; (2) PIP levels should associate with ocular surface microbiome composition (if sampled) or with clinical signs of blepharitis/meibomian gland dysfunction; and (3) anti-inflammatory or microbiome-targeted interventions that normalize ocular surface inflammation should partially restore PIP if this mechanism is dominant.

Finally, an essential “mechanism” to test—because it can masquerade as biology—is pre-analytical and analytical modulation, especially given that PIP is glycosylated and tear sampling methods can alter the measured proteome. Tear collection approaches (Schirmer strip vs. capillary tubes), reflex tearing, and protein extraction protocols change relative abundances for many proteins, and the tear proteomics field increasingly emphasizes method standardization and cross-platform validation [3,46,78,79]. If PIP is particularly sensitive to extraction efficiency, adsorption, or proteolysis, cohort differences could be exaggerated. Testable predictions will be that (1) PIP differences should replicate across collection methods and platforms (targeted MS and immunoassays); (2) spiked recovery experiments should show comparable extraction across AD/control tear matrices; and (3) PIP decreases should persist after normalizing to tear volume/total protein and after accounting for reflex tearing indicators.

**Figure 1:**
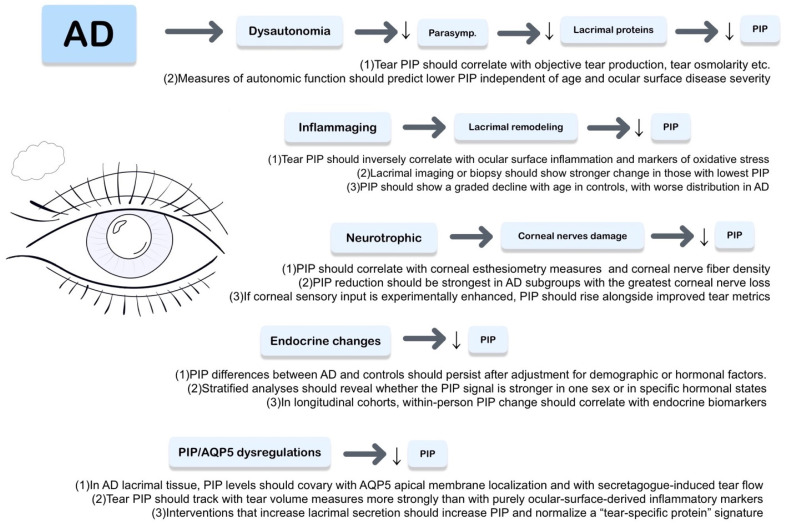
Hypothetical mechanisms of AD-associated tear PIP reduction with testable predictions.

Taken together, the most biologically coherent model suggests that the reduction in tear PIP in AD may result from a combination of LFU dysregulation (sensory neuropathy + autonomic/cholinergic imbalance), lacrimal gland secretory remodeling (inflammaging/oxidative stress), and potentially AQP5 trafficking disturbances in exocrine epithelia—with secondary contributions from endocrine and immune/microbiome shifts. The advantage of framing the problem mechanistically is that it turns “PIP is down” into a set of concrete experiments: correlate PIP with tear volume and corneal nerve metrics, connect it to AQP5 localization and secretagogue responsiveness, and test whether the signal survives rigorous method controls and ocular surface phenotyping. These hypothetical mechanisms and their corresponding testable predictions are summarized in Figure 1.

## 7. Is PIP a Good AD Biomarker?

Whether PIP is a good AD biomarker depends on the benchmark being applied. In modern biomarker evaluation, the question is typically decomposed into analytical validity (can it be measured precisely and reproducibly?), clinical validity (does it distinguish AD or AD biology from relevant comparators?), and clinical utility (does it improve decisions or outcomes in a defined use case?). Diagnostic-accuracy reporting standards (STARD 2015) emphasize that an index test must be evaluated against an appropriate reference standard in a population matching the intended use, with transparent reporting of sampling, blinding, missingness, and prespecified analyses—requirements that tear biomarkers for AD are only beginning to meet at scale [80,81].

From the standpoint of clinical validity, PIP has a notable advantage: it is one of the relatively few tear proteins for which multiple AD tear studies converge on the same direction of effect—lower tear PIP in AD. The clearest quantitative early evidence comes from Kalló et al. (2016) [15], who used targeted SRM quantification of a set of abundant tear proteins and reported a significant decrease in PIP in AD alongside decreases in several lacrimal/tear-specific proteins (with dermcidin increased), and then explored multivariate combinations for discrimination. More recently, Kärkkäinen et al. (2025) [17] reported, in a neuroinflammation-focused tear proteomics study of mild AD, that PIP was one of only two significantly downregulated proteins among a set of inflammation-related candidates, again consistent with a “tear-specific proteins down + inflammatory proteins up” pattern. In 2026, an additional tear proteomics report in MCI described altered tear protein expression with patterns reported as similar to those in AD, supporting the broader idea that tear proteome remodeling begins early on the cognitive-impairment spectrum; this sort of staging relevance is important if PIP is to be useful for screening rather than end-stage classification [82]. In light of the above, we suggest that future studies should include other types of dementia, as well as account for age, in order to determine to what extent the observed PIP signal can be distinguished from changes occurring in other forms of dementia and whether it is specific to Alzheimer’s disease or instead reflects broader alterations across the spectrum of cognitive disorders.

The key limitation for PIP as an AD biomarker is specificity. Specificity can be assessed by examining how PIP behaves across various conditions underpinned by aging, inflammation, or neurodegeneration. Current findings suggest that PIP does not show a behavior unique to AD. In other neurodegenerative diseases, such as Parkinson’s disease, untargeted proteomic profiling of tears has identified downregulated tear proteins linked to immune response, inflammation, apoptosis, and lysosomal function, indicating that a disease-associated “tear signature” is not unique to AD [83]. Moreover, the direction of PIP change is not consistent across diseases. Proteomic analysis of the tear film in patients with age-related macular degeneration showed that PIP, along with many other proteins, was upregulated [84]. A similar situation was observed in patients with dry eye disease, in whom PIP levels were likewise elevated [43]. Taken together, these observations suggest that tear PIP levels vary depending on a range of conditions that are common in the elderly population. This considerably limits the interpretation of low PIP levels in AD, meaning that a decreased PIP concentration cannot definitively be regarded as an event specific to AD. Tears are not a “brain-specific” biofluid; tear composition is strongly shaped by ocular surface state, lacrimal gland function, sampling method, and systemic inflammation. TFOS DEWS II emphasizes that DED is characterized by tear film instability and compositional shifts, including protein changes, and it documents that standard clinical tests (e.g., Schirmer) have variability and must be interpreted in context [85,86]. Because PIP is a tear/lacrimal-associated protein, and because AD cohorts are typically older (with high background prevalence of DED, meibomian gland dysfunction, medication use, and reduced blink/ocular surface exposure), an observed PIP decrease can reflect at least two non-exclusive realities: (i) an AD-linked LFU change (autonomic/neuroimmune), or (ii) a higher burden of ocular surface dysfunction in the AD group. This makes PIP more credible as a “lacrimal homeostasis/tear-specific” marker than as an AD-specific marker by itself, even if its direction of change is consistent. It appears essential for future studies to implement rigorous exclusion criteria, with particular emphasis on the presence of active ocular surface disease, recent infections, severe autoimmune sicca syndromes, and recently initiated anti-inflammatory therapy. Alternatively, at a minimum, participants should be stratified according to the presence of DED, which could help mitigate the influence of confounding factors on the interpretation of the findings.

A second limitation is that the current tear PIP literature is still relatively small and cohort-specific, which makes it hard to estimate real-world performance metrics with confidence. Kalló et al. reported diagnostic performance primarily for multi-protein panels (rather than claiming PIP alone is definitive), and that is a strength of their interpretation: the best discrimination came from combinations, consistent with the idea that AD tears reflect a pattern rather than a single molecule. Kärkkäinen et al. similarly position PIP as part of a tear signature rather than as a lone classifier, and their focus on mild AD is clinically relevant but still needs replication across sites and platforms. In biomarker science, early strong *p*-values do not guarantee robust classification across populations with different comorbidities and collection conditions; STARD exists largely because the literature repeatedly overestimates diagnostic performance when designs are not aligned with intended use and when confounders are not rigorously controlled.

In that respect, it is useful to compare PIP with the parallel tear–AD biomarker track focused on canonical AD analytes (amyloid and tau) and on multimodal validation designs. A proof-of-concept tear study in 2019 reported differential tear proteins and microRNAs across AD/MCI/controls, showing that tears can carry disease-associated molecular differences beyond abundant lacrimal proteins, but it also underscores that tear biomarkers can be numerous and that selection/validation is critical [16]. A 2021 *Scientific Reports* study linked tear tau measures to neurodegeneration categories and disease severity, illustrating how tear biomarkers can be anchored to established AD biology rather than to lacrimal physiology alone [60]. The TearAD project (protocol and rationale published in 2023) explicitly aims to validate tear amyloid/tau measures and evaluate diagnostic accuracy against neurodegeneration-related outcomes (e.g., hippocampal atrophy), which is closer to the kind of reference-standard anchoring that would strengthen any tear biomarker, including PIP, if incorporated into a composite panel [19]. Reviews of tear biomarkers in AD similarly frame abundant tear proteins (including PIP in the older tear-proteome studies) as candidates that may contribute to a panel but highlight the need for larger, standardized validation [62].

From an analytical validity perspective, PIP is actually fairly well positioned relative to many low-abundance tear analytes: it is a known tear constituent and can be measured by multiple orthogonal methods (targeted MS, immunoassays). The existence of both targeted SRM quantification (2016) and later label-free proteomics (2025) pointing in the same direction is encouraging because it reduces the likelihood that the entire signal is an artifact of one platform. That said, tear sampling and processing still impose major pre-analytical variance, and TFOS DEWS II methodology discussions emphasize that tear tests and tear composition can vary with reflex tearing and collection conditions; therefore, PIP’s analytical robustness must be demonstrated under the exact sampling approach proposed for clinical use (e.g., Schirmer strip extraction protocol). Particular attention should be paid to the method of tear collection, storage conditions including factors such as temperature, and normalization procedures. Efforts should be made to minimize the impact of technical variability on the final interpretation of the measured concentrations. The growth of dataset deposition and integrated records for tear–AD studies (e.g., BioStudies/OmicsDI records linked to tear proteomics and microRNA publications) helps, because it allows independent re-analysis and harmonization attempts—an increasingly important expectation for biomarker translation [16].

This raises the possibility of considering PIP concentrations not only in tears but also in other biological fluids. Although studies of cerebrospinal fluid and blood in Alzheimer’s disease have identified numerous potential biomarkers, to the best of our knowledge the available literature does not appear to contain a well-developed body of work specifically focused on PIP in cerebrospinal fluid, serum, or plasma.

Considering clinical utility, the most realistic near-term use-case for PIP is not “diagnose AD” but “help triage or stratify” when combined with other tear markers. In practice, a tear-based screening tool will likely need to (a) be inexpensive and scalable, (b) enrich for individuals who should receive confirmatory CSF/PET or advanced plasma biomarkers, and (c) remain stable enough across common ocular surface comorbidities to avoid unacceptably high false positives. This logic is explicitly reflected in TearAD’s motivation: tear biomarkers could serve as a front-line screener if they meaningfully predict neurodegeneration-related outcomes and/or AD biomarker positivity. Under that framework, PIP’s value is most plausibly as a component that captures the lacrimal/tear homeostasis axis (which seems to shift in AD) while amyloid/tau or inflammation-linked markers capture more disease-specific biology; combining “tear-specific proteins down” with “AD biology up” may produce a classifier that is both sensitive and more specific than either class alone. The 2016 tear biomarker modeling already supports the broader principle that combinations outperform single markers in tears.

### Recommendations to Mitigate Confounding in Tear PIP Studies

- Inclusion/exclusion criteria: persons with active ocular surface disease, autoimmune sicca syndromes (e.g., Sjögren’s syndrome), recent ocular surgery, or recent use of anti-inflammatory medications should be excluded.
- Ophthalmological characterization: A standardized ophthalmological assessment should be implemented to ensure appropriate stratification of patients enrolled in the study. Clinical phenotyping of the ocular surface in all participants meeting the inclusion criteria also appears essential.
- Comparator groups: future studies should ensure appropriate matching for age and sex within a cognitively unimpaired control group, as well as the inclusion of at least one group of patients with a non-AD dementia, which would substantially open the way for a rigorous assessment of the specificity of this marker with respect to the changes observed in AD.
- Sample collection: a single, well-defined collection method should be specified and consistently implemented (Schirmer strip vs. microcapillary), given the variability in the proteomic profiles obtained.
- Standardization of pre-analytical control: a uniform extraction protocol, sample storage temperature, and the number of freeze–thaw cycles must be defined.
- Analytical validation: the PIP signal should be confirmed using at least two independent yet complementary analytical methods.

## 8. Conclusions

PIP is a promising but limited AD tear biomarker on its own. Its strengths are (i) repeated observation of decreased levels in AD tears across distinct study designs, (ii) biological plausibility as a lacrimal/tear homeostasis protein, and (iii) measurability by orthogonal platforms. Its weaknesses are (i) likely limited specificity because many ocular surface and systemic conditions can modulate tear composition, (ii) insufficient large multi-center validation with rigorous reference standards aligned to intended use, and (iii) vulnerability to pre-analytical variation inherent to tear sampling and tear film instability, particularly in older populations. In other words, PIP is best viewed today as a panel feature (and potentially a stratifier of lacrimal dysfunction phenotypes within AD cohorts) rather than as a stand-alone diagnostic marker for AD.

## Figures and Tables

**Table 1:** Summary of key tear studies.

Study	Year	Cohort	Methods	Tear Level of PIP	Key Findings
Kalló et al. [15]	2016	23 (14 AD, 9 controls)	Tear samples were collected using microcapillary tubes; the global protein profile was analyzed by SDS-PAGE followed by LC-MS/MS, and subsequently quantified using targeted SRM	Decreased in patients with AD	PIP levels, along with other proteins secreted by the lacrimal gland, are decreased, suggesting lacrimal gland dysfunction in patients with AD
Kärkkäinen et al. [17]	2025	53 (19 AD, 34 controls)	Tear samples were collected using Schirmer strips; the protein profile was analyzed by LC-MS/MS with label-free quantitative analysis	Decreased in patients with AD	PIP levels, along with other proteins secreted by the lacrimal gland, are decreased, suggesting lacrimal gland dysfunction in patients with AD
Gijs et al. [60]	2021	65 (23 SCD, 22 MCI, 11 dementia 9 controls)	Tear samples were collected using Schirmer strips; direct tear film immunoassay analysis was performed.	No direct measurement	This supports the concept that tear film composition may reflect degenerative changes in the CNS

Abbreviations: Alzheimer’s Disease (AD); Sodium Dodecyl Sulfate–Polyacrylamide Gel Electrophoresis (SDS-PAGE); Liquid Chromatography–Tandem Mass Spectrometry (LC-MS/MS); Selected Reaction Monitoring (SRM); Prolactin-Inducible Protein (PIP); Subjective Cognitive Decline (SCD); Mild Cognitive Impairment (MCI); Central Nervous System (CNS).

**Table 2:** Tear biomarker candidates in AD.

Analyte Class	Representative Analytes	Biological Axis	Strengths	Limitations	Reference
Lacrimal gland-linked proteins	Lipocalin-1; lactotransferrin; lacritin; lysozyme-C	LFU	Candidate markers of AD-associated LFU secretory dysfunction	Limited clinical validation; Reduced specificity for AD; High susceptibility to confounding factors	[15]
Canonical AD molecules	Amyloid-β; Tau	AT(N) system	Direct brain pathology link; reported associations between analytes levels in tears and disease severity	No established tear-fluid cut-off values; Small sample volumes require dilution/preprocessing	[60]
microRNA	microRNA-200b-5p	Gene regulation, protein expression	High detectability in tear fluid; increased total microRNA concentration in AD vs. controls	Limited clinical validation; Unclear mechanistic link to AD; High susceptibility to confounding factors; No established tear-fluid cut-off values	[16]
Other tear-fluid proteins	16 proteins (e.g., NP1L4; BBOX1; CYTC; HNRNPA2B1; ERO1α)	Heterogeneous: oxidative stress, protein synthesis, energy metabolism	May reflect diverse pathophysiological processes in AD; Some analytes previously linked to AD (e.g., CYTC)	Limited clinical validation; Unclear mechanistic link to AD	[18]

Abbreviations: Alzheimer’s Disease (AD); Lacrimal Functional Unit (LFU); Nucleosome assembly protein 1-like 4 (NP1L4); Gamma butyrobetaine dioxygenase (BBOX1); Cystatin C (CYTC); Heterogenous nuclear ribonucleoprotein A2/B1 (HNRNPA2B1); ERO1-like protein alpha (ERO1α).

## Data Availability

No new data were created or analyzed in this study. Data sharing does not apply to this article.

## Abbreviations

The following abbreviations are used in this manuscript:

AD	Alzheimer’s disease
DED	Dry eye disease
PIP	Prolactin-inducible protein
GCDFP-15	Gross cystic disease fluid protein-15
SABP	Secretory actin-binding protein
EP-GP	Extraparotid glycoprotein
AZGP1/ZAG	Zinc-alpha-2-glycoprotein
HAP	Human Protein Atlas
AQP5	Aquaporin-5
SS	Sjögren’s syndrome
SRM	Selected reaction monitoring
LFU	Lacrimal functional unit
HRV	Heart-rate variability
